# *Lactococcus lactis* as an Effective Mucosal Vaccination Carrier: a Systematic Literature Review

**DOI:** 10.4014/jmb.2411.11036

**Published:** 2025-03-07

**Authors:** Suryanata Kesuma, Tri Yudani Mardining Raras, Sri Winarsih, Takeshi Shimosato, Valentina Yurina

**Affiliations:** 1 Doctoral Program in Medical Science, Faculty of Medicine, Universitas Brawijaya, East Java 65145, Indonesia; 2 Departement of Medical Laboratory Technology, Poltekkes Kemenkes Kalimantan Timur, Samarinda, East Borneo; 3 Departement of Biochemistry and Molecular Biology, Medical Faculty, Universitas Brawijaya, Malang 65145, Indonesia; 4 Department of Pharmacy, Medical Faculty, Universitas Brawijaya, Malang 65145, Indonesia; 5 Institute for Aqua Regeneration, Shinshu University, Nagano, Japan

**Keywords:** *Lactococcus lactis*, cellular immunity, humoral immunity, mucosa, vaccine, vaccine evaluation

## Abstract

*Lactococcus lactis* has potential as a mucosal vaccine delivery system. *L. lactis* can express antigens from bacteria or viruses, which are tightly controlled using nisin. Although *L. lactis*-based vaccine shows great promise, no product is ready for human use. Several studies have been conducted to develop *L. lactis*-based vaccine, and the efficacy of these vaccines has been evaluated in many scientific articles. This paper aims to review key aspects of current knowledge on the promising characteristics of *L. lactis* and to suggest its implications for vaccine design. Articles were obtained online using inclusion and exclusion criteria through Harzing's Publish or Perish. The article assessment used the Joanna Briggs Institute critical appraisal checklist for quasi-experimental studies. The efficacy evaluation of 24 articles showed that *L. lactis*-based vaccine can induce IgA and IgG as humoral immune responses; T CD4, T CD8, and B cells as cellular immune responses; and various proinflammatory cytokines such as IFN-γ, TNF-α, IL-2, IL-4, IL-8, IL-10, IL-12, IL-17. *L. lactis* is suitable as a vector carrier for oral or nasal mucosal vaccines targeting bacterial and viral infections. The development of *L. lactis* as a vaccine delivery system is promising.

## Introduction

Lactic Acid Bacteria (LAB) are an excellent candidate for manipulation as a mucosal vaccine carrier. LAB is resistant to acidic conditions in the gastrointestinal system and can effectively deliver vaccines to the intestinal area. One of the LAB widely applied as a carrier vaccine is *Lactococcus lactis* (*L. lactis*). Naturally, *L. lactis* enhances the immune response to pathogens by inhibiting their colonization in the gastrointestinal tract and boosting the immunological system of the mucous membrane intestine [1]. The ability of *L. lactis* to pass through the intestinal tract without colonization, its Gram-positive status (it does not contain endotoxins), its safety for consumption, genetic material is easy to manipulate, its ease of handling, its rapid growth, its ability to express stable recombinant proteins (antigens), and its low production costs due to the lack of protein purification are further significant benefits of using *L. lactis* as a mucosal vaccine carrier [2, 3]. In addition, peptidoglycan in *L. lactis* has benefits as an adjuvant, in addition to being a location for antigen expression, this peptidoglycan can bind to various pattern recognition receptors (PRRs) [4]. Peptidoglycan can interact with Toll-Like Receptors (TLR2), NOD-Like Receptors (*e.g.*, NOD1 and NOD2), C-Type Lectin Receptor (*e.g.*, Dectin-1) so that it will trigger an innate immune response to *L. lactis*-based vaccine [5, 6].

*L. lactis* as a mucosal vaccine carrier is called *L. lactis*-based vaccine can overexpress antigen using the NICE (nisin-controlled gene expression) system to control their protein expression [7]. The mechanism of nisin induction in the NICE system involves the histidine kinase NisK, which captures the nisin-induced signal and undergoes autophosphorylation, transferring the phosphate group to the NisR response regulator protein, thereby activating the NisA promoter [8, 9]. *L. lactis*-based vaccine can stimulate the immune response when administered orally or nasally. When administered orally, *L. lactis*-based vaccine will go to the gut, Peyer's patches [10]. On the intestine, M cells transport the antigen carried by *L. lactis* through the lumen epithelium of intestine via a transcytosis mechanism to the dendritic or antigen-presenting cells (APCs) in the space between Peyer's patches, known as the intrafollicular region (IFR). APCs then present the antigen peptides to B and T lymphocytes to induce an adaptive immune response [11].

As previously described, there are many benefits of using *L. lactis*, especially as a as a mucosal vaccine carrier and producer of recombinant proteins such as antigens that can activate innate and adaptive immune responses. This research may facilitate future scientific advancements on utilizing *L. lactis* as a mucosal vaccine carrier/*L. lactis*-based vaccine. We believe conducting further research on *L. lactis* as a vaccine delivery system is essential. This paper aims to review critical points of current knowledge on the promising characteristics of *L. lactis*-based vaccine to suggest its implications for vaccine design.

## Materials and Methods

The descriptive research uses a systematic literature review (SLR) methodology. The systematic literature review was informed by data extrapolated from a concurrent comprehensive analysis, which sought to integrate and appraise the implementation of *L. lactis* as a vector for mucosal vaccine delivery systems or called *L. lactis*-based vaccine. The search and selection of literature, in the form of scientific articles, followed the Preferred Reporting Items for Systematic Literature Reviews and PRISMA protocol [12].

### Identification Strategy

The article search was conducted in three databases, namely Crossref and PubMed, using Harzing's Publish or Perish application with keywords such as "*Lactococcus lactis*" OR “*L. lactis*” AND "vaccine" OR “Vaccines” and "immunity" AND “mucosal" OR “Mucosal”.

### Study Selection

Articles had to meet the following inclusion criteria: 1) research articles published in the last ten years (2013–2023); 2) English-language articles indexed in the databases used; 3) true experimental studies; 4) original articles; 4) studies meeting PICO criteria (population: research using *L. lactis* to enhance the immune response; intervention: giving recombinant *L. lactis* on trial *in vitro* and *in vivo*; comparison: animal trial without treatment (control); giving *L. lactis* without gene insert; outcome: improving the immune system; 5) vaccines for infectious diseases; 6) critical evaluation score of ≥ 50%. In contrast, the exclusion criteria included: 1) a review article, thesis, or protocol; 2) an *in-silico* study; 3) combination of adjuvants; 4) articles not available in full text; 5) studies not related to vaccine delivery system 6) studies focusing only on a probiotic; 7) vaccines for animals; 8) clinical trials; 9) non-living bacteria delivery system.

### Data Assessment

Data quality analysis was conducted using critical evaluation tools, specifically the Joanna Briggs Institute critical appraisal checklist for quasi-experimental studies. The checklist consists of 9 questions, where a "yes" answer is worth 1 point, and "no," "unclear," or "not applicable" answers are valued at 0 points. The results of this analysis are supported by the quality analysis of the journals, considering their quartile rankings and impact factor values [13, 14].

### Data Analysis

Descriptive statistical methods were employed to encapsulate the research attributes incorporated within this systematic review. The data is presented using Microsoft Excel, VOSviewer and R Studio.

## Results

The initial search resulted in 2729 articles discussing using *L. lactis* as a mucosal vaccine carrier. After removing duplicates, 1883 articles remained. A quick screening of the title and abstract reduced this number to 146 articles. Further screening against the exclusion criteria resulted in 24 articles. All articles were assessed using the Joanna Briggs Institute critical appraisal, and it was determined that 24 articles were included in the analysis of this study.

Fig. 3 describes *L. lactis*-based vaccine. Mice are preferred as experimental animals because they are easier to handle. The antigen protein expression target location is extracellularly preferred by adding signal peptides because the immune system can directly recognize and represent the antigen. *L. lactis*, as a mucosal vaccination carrier, can also be combined with additional adjuvants. When challenged, the author also reported protection in experimental animals against bacterial or viral infections.

## Discussion

**Fig. 1:**
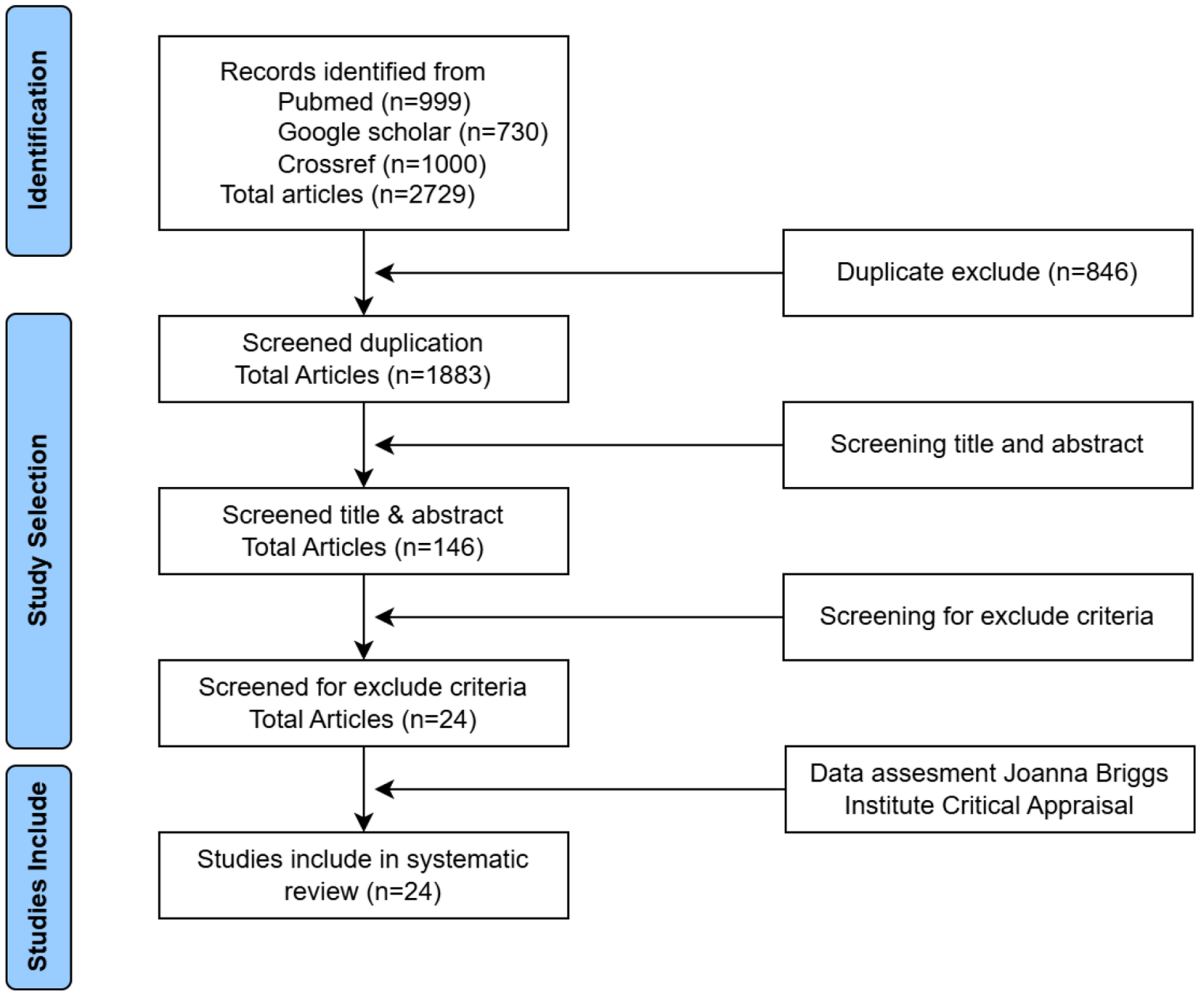
PRISMA flow diagram for study selection.

The results of screening in PubMed, CrossRef, and Scholar databases using the corresponding keywords yielded a total of 2,729 articles. An initial analysis was conducted to eliminate duplicate titles reducing the total number to 1,883 articles. Further analysis was conducted to select titles and abstracts articles, narrowing down the selection to 146 articles available for full access. Additional exclusions were made based on content relevance, resulting in a final selection of 24 articles for analysis (Fig. 1).

*L. lactis* is a nonpathogenic Gram-positive bacterium widely used in the dairy industry. *L. lactis* has been widely explored for its potential as a vector for delivering therapeutic molecules such as vaccine antigens. The application of *L. lactis* as a mucosal vaccine delivery system has been widely investigated over the past two decades, demonstrating its versatility in expressing heterologous proteins, cytokines, and enzymes. *L. lactis* has been proposed as a safe platform for the mucosal vaccine carrier, and it can be genetically modified to express specific antigens on its surface, intracellularly, or extracellularly [39].[Fig F2]

*L. lactis* has a good safety history because of its benefits in food fermentation. It survives in the digestive tract for 2-3 days and does not attack the intestinal mucosal surface [40]. *L. lactis* does not have lipopolysaccharides, so it will not strongly stimulate the host's immune response, so it is safe when given repeatedly [3, 24, 41]. *L. lactis* also possesses immunomodulatory abilities as a probiotic bacterium. It can enhance the activity of phagocytic cells, which engulf and destroy pathogens [42, 43]. Expression of recombinant proteins such as antigens is very effective in *L. lactis*. This is due to the tight control with the addition of the inducer Nisin [31]. Antigens can be expressed intracellularly, extracellularly, or on the surface of *L. lactis* by adding signal peptides to the gene of interest [19, 28]. Antigens expressed intracellularly require immune cells to degrade or phagocytose *L. lactis*-based vaccine first to uptaken the antigen. This differs from antigens expressed extracellularly or on the surface of *L. lactis*-based vaccine, immune cells can directly recognize antigens so that the immune response of memory or antibodies to antigens can be produced faster [24, 32]. In addition, *L. lactis*-based vaccine also stimulate the production of proinflammatory cytokines, which are essential molecules in regulating immune responses and inflammation. Among the cytokines produced with the help of *L. lactis* are IFN-γ and TNF-α. IFN-γ plays a significant role in activating immune cells, such as macrophages and T cells, which combat infection. TNF-α is also essential in mediating the inflammatory response that helps to destroy pathogens and repair damaged tissues [44-46].

### Protein Expression System and Location

The Nisin Controlled Gene Expression (NICE) system employed by *L. lactis* is a highly effective and easy to use gene expression method. The procedure involves introducing a specific quantity of the Nisin inducer (0.1-5 ng/ml) into the growth medium [47]. The NICE system operates through a signal transduction mechanism involving two primary proteins, NisK and NisR. NisK is a sensor protein located on the membrane, while NisR is a response regulator protein in the cytoplasm. Nisin as an inducer can interact with NisK, causing it to undergo autophosphorylation. The phosphate group is then transferred to NisR, activating it. NisR initiates transcription of the target gene on the plasmid by binding to the downstream part of the PnisA promoter. The uniqueness of the NICE system lies in its ability to control protein expression with high precision, allowing for efficient and controlled protein production. This mechanism makes *L. lactis* a valuable tool in biotechnology research and applications, particularly in vaccine production [7, 47-49].

**Fig. 3:**
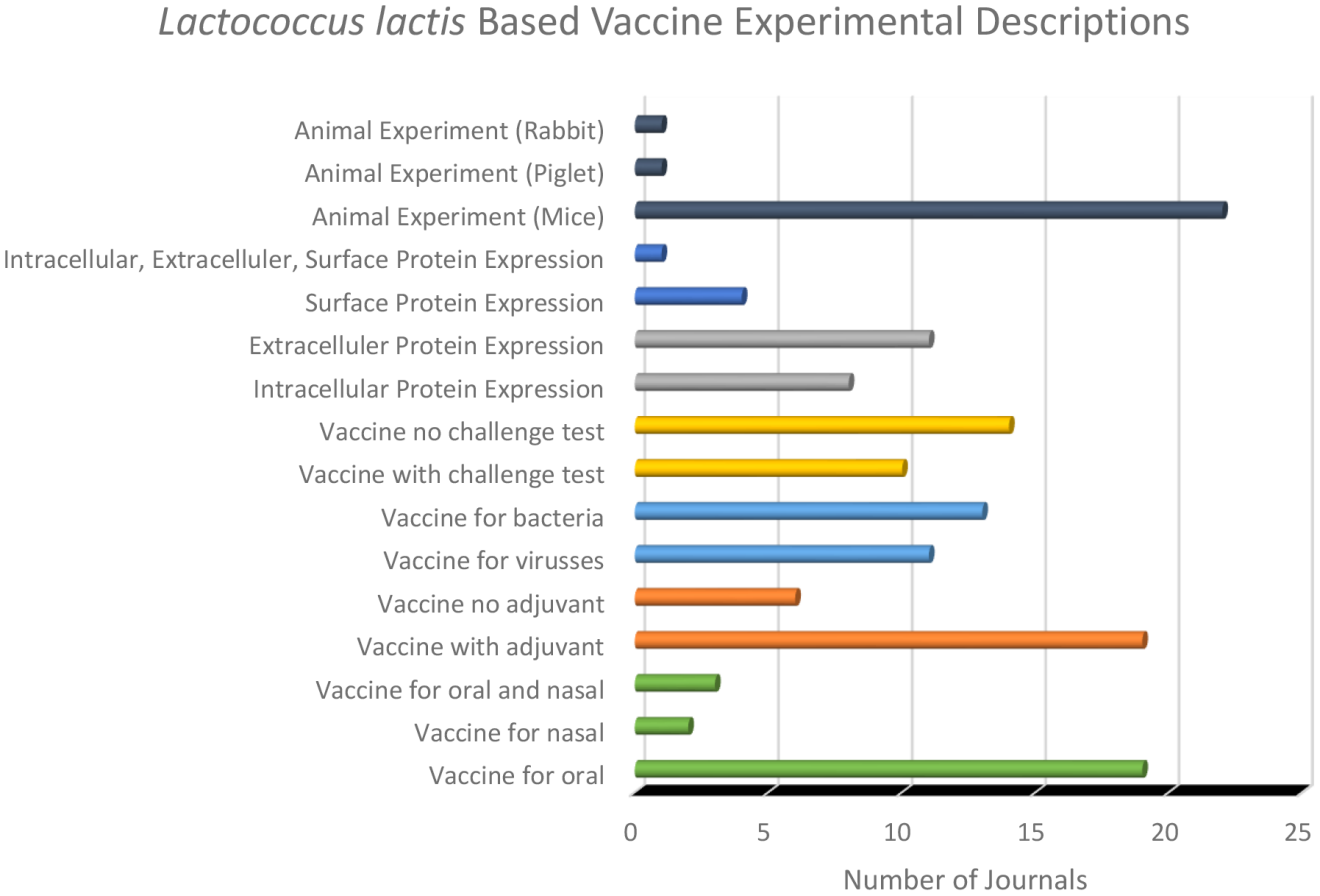
*L. lactis* Based Vaccine Experimental Descriptions.

*L. lactis*-based vaccine can be expressed as cell surface antigen. One method for designing the gene of interest involves adding a signal peptide, a technique extensively explored for its potential in developing *L. lactis*-based vaccine. The studies in Table 1 and Fig. 3 demonstrate the successful antigen expression on the surface of *L. lactis*, which can stimulate immune responses [19, 21, 23, 36]. The pgsA signal peptide is particularly effective for expressing proteins on the surface of *L. lactis*. The pgsA protein facilitates protein translocation across the cytoplasmic membrane, allowing for effective secretion into the extracellular space or anchorage to the cell wall [50]. Several studies in Table 1, such as those by [23], have successfully utilized the pgsA signal peptide in *L. lactis*/pNZ8110-pgsA-NA that has been constructed, highlighting its critical role in facilitating protein display on the surface of *L. lactis*-*based* vaccine.

Fig. 3 and Table 1 also report several studies concerning extracellular protein expression in *L. lactis*. The signal peptide used for extracellular protein expression differs from the signal peptide used for protein expression on the cell surface. The USP45 signal peptide has been employed to enable the secretion of target proteins by *L. lactis* [32]. The Usp45 signal peptide is added to the N-terminus of the target protein to facilitate its secretion by *L. lactis* [28]. In the study by [26], the pNZ8124:sip vector containing the lactococcal Usp45 signal peptide sequence (SP usp45) fused to the PnisA promoter was successfully constructed, allowing the target protein to be expressed extracellularly by *L. lactis*.

[17] reported the successful utilization of the Usp45 signal peptide for the extracellular expression of the *Helicobacter pylori* Lpp20 antigen using *L. lactis*. This study also demonstrated that extracellular vaccination with *H. pylori* Lpp20 was more effective. Other researchers, such as [15, 22, 25, 30-32, 34, 35, 37] have also proven that the usp45 signal peptide can be used to express extracellular recombinant proteins in *L. lactis*.

### *Lactococcus lactis* Strain

Several strains of *L. lactis* are commonly used in vaccine delivery systems. Table 1 shows that *L. lactis* strains NZ9000 and NZ3900 are widely used as mucosal vaccine carriers. Both strains cannot grow on media containing only lactose as a carbon source due to the deletion of the LacF gene, necessitating a plasmid carrying the LacF gene operon. The LacF gene detection system is a selection system that determines whether cells carry the plasmid or not. In addition, both strains have the PnisA promoter, allowing for tightly controlled protein expression [7, 47, 51].

Table 1 and Fig. 1 show that *L. lactis* NZ9000 has been used to express viral proteins, bacterial antigens, and fusion proteins. This demonstrates its versatility in vaccine development. *L. lactis* NZ9000 has been used as a live bacterial vaccine platform to present antigens from pathogens such as Group A *Streptococcus*, *Helicobacter pylori*, and *influenza* [16, 18, 52]. In addition, *L. lactis* NZ9000 has been used to express antigens from pathogens such as *Brucella melitensis* [34], *Human papillomavirus* [22], *Hepatitis* A VP1-P2a antigen [38] and Neuraminidase protein from *Influenza A* [39] demonstrating its potential in developing vaccines against viral infections. This strain can deliver antigens to mucosal sites and induce mucosal and systemic immune responses. One of the advantages of using *L. lactis* NZ9000 is its ability to stimulate both humoral and cellular immune responses. Studies in Table 1 have shown that oral and mucosal immunization with *L. lactis* NZ9000 expressing specific antigens can elicit strong antibody responses, including IgG and IgA, and activate T cells, which promote a strong immune response against a variety of pathogens.

In addition to the NZ9000 strain, *L. lactis* NZ3900 has also been widely used in the development of *L. lactis*-based vaccine, as shown in Table 1. *L. lactis* NZ3900 has been genetically engineered to maximize the expression of vaccine proteins. For example, [27] demonstrated that *L. lactis* NZ3900 was used to deliver the Highly Conserved Region Spike S2 antigen for oral and nasal immunization in BALB/c mice. Other studies have also reported success in developing *L. lactis*-based vaccine that express bacterial or viral antigens in *L. lactis* NZ3900, such as antigens from *H. pylori* [15, 17, 31] pertussis toxin and filamentous hemagglutinin from *Bordetella pertussis* [25], antigens from Enterotoxigenic *Escherichia coli* [32] and M-protein antigens derived from Group A *Streptococcus pyogenes* [33]. This shows that this strain can express antigens from bacteria or viruses.

### Doses and Route

Dosage is a critical component in vaccine development and administration. Dosage is crucial as it ensures the vaccinés efficacy and safety. The vaccine dose determines the amount of antigen given to the body to trigger a strong immune response with the least side effects. Dosage determination begins with preclinical studies, followed by several phases of clinical trials [53-55].

Table 1 shows that the dose of *L. lactis*-based vaccine is measured in colony forming units (CFU) and can be adjusted according to the experimental animals used besides determining the number of CFU [37, 56]. Dosages for *L. lactis*-based vaccine can start from as low as 10^6^ CFUs and can range up to 10^9^ CFUs or more, depending on the immunogenicity of the antigen and the delivery method [57, 58] Vaccination with a prime-boost strategy can also be applied, initial dose (prime) is followed by one or more subsequent doses (boost) to increase the immune response. The interval and frequency between doses and the total dose are also important factors. The interval and frequency of vaccination can range from a few weeks to several months to build a stronger and more durable immune response [26, 59, 60, 61]. Moreover, the route of administration plays a role in determining the dosage. Oral administration might require higher doses than nasal administration due to the degradation of bacteria in the gastrointestinal tract [27, 62]. Stabilizers and adjuvants are often included to protect the bacteria and enhance the immune response [63].

Based on Table 1, in various experimental animal models other than mice, larger doses are generally observed; for instance, piglets receive doses ranging from 10^9^ to 10^12^ CFU [16] and rabbits receive 5 × 10^9^ CFU [32]. In contrast, vaccine doses administered to mice ranged from 1x10^8^ CFU [20] to 5 × 10^14^ CFU [17]. The route of administration also influences the dosage of *L. lactis*-based vaccine, with the oral route requiring higher doses of approximately 5 × 10^9^ CFU compared to the nasal route, which utilizes doses around 1 × 10^9^ CFU [27].

The dose of a *L. lactis*-based vaccine administered via the oral route tends to be higher than that given nasally due to several factors related to the body's immune system and physical barriers in the gastrointestinal (GI) tract. The first factor is exposure to digestive enzymes and the harsh environment of the GI tract. The oral route exposes the vaccine to acidic pH and digestive enzymes like pepsin and proteases, which can degrade the *L. lactis*-based vaccine and reduce its efficacy. Therefore, a higher dose is needed to ensure enough *L. lactis*-*based* vaccine survive to stimulate an immune response [27, 64]. The second factor is the immune system's complexity in the gut-associated lymphoid tissue (GALT), which includes structures like Peyer's patches that efficiently sample and respond to antigens. Additionally, normal intestinal flora competes with and may neutralize *L. lactis*-based vaccine. These two main factors necessitate higher doses for oral [65-67].

*L. lactis*-based vaccine administered nasally is directly exposed to the nasal-associated lymphoid tissue (NALT). The nasal mucosa, especially NALT, is more efficient at antigen uptake, resulting in a strong immune response. This allows for a lower dose of *L. lactis*-based vaccine than oral administration [68].

### Experimental Animals

Experimental animals are essential in preclinical studies for developing *L. lactis*-based vaccine. Commonly used experimental animals include rats and mice, chosen for their physiological and immunological similarities to humans. These animals are relatively inexpensive and easy to obtain. Preclinical studies on these animals are critical for assessing vaccine safety, immunogenicity, and efficacy [26, 69].

The study in Fig. 3 shows that 92% of the studies used mice (specifically BALB/c, C57BL/6, and FVB/n strains), while the remaining studies employed piglets and rabbits. Specific inbred strains such as BALB/c and C57BL/6 mice are preferred within these species. Inbred strains ensure genetic uniformity, reducing variability in immune responses and improving reproducibility of results.

### Vaccine Evaluation

The efficacy of *L. lactis*-based vaccine can be measured by assessing the immune response they produce. The primary immune response in *L. lactis*-based vaccine is the mucosal immune response, which can be assessed by measuring IgA or IgG antibodies circulating systemically. The levels of these antibodies indicate the strength of the humoral immune response triggered by *L. lactis*-based vaccine [19, 25, 32]. Table 1 shows that all studies related to *L. lactis*-based vaccination reported a significant increase in humoral immune responses. The parameters for assessing humoral immune responses include IgG and IgA antibodies, with IgG evaluation generally performed on serum and IgA assessment on feces, tissues (intestine, colon, nose), and nasal fluid. In addition to antibodies, the assessment of cellular immune responses is crucial for evaluating the efficacy of *L. lactis*-based vaccine. This can be done by measuring the activation of T cells, especially CD4 T cells and CD8 T cells, which are essential in coordinating the immune response and directly targeting infected cells [24, 29]. In addition to humoral and cellular immune responses, the efficacy of *L. lactis*-based vaccine can be assessed by measuring the cytokine profile. One common cytokine measured is interferon-gamma (IFN-γ). IFN-γ, which can provide an overview of the polarization of CD4 T cell responses, including Th1 cells pathways [20, 25]. Table 1 shows that several researchers have reported a significant increase in IFN-γ in experimental animals immunized with *L. lactis*-based vaccine. As previously mentioned, IFN-γ analysis in the study [20] was used to assess Th1 cell activation. Activated Th1 cells will release IFN-γ to help activate cytotoxic T cells, NK cells, and macrophage cells. As a step to initiate the immune response, *L. lactis*-based vaccine directly interacts with the mucosal surface of the gastrointestinal tract or nose, depending on the route of administration. The mucosal surface is rich in Microfold (M) and dendritic cells as an *Antigen Precenting Cell* (APC). Microfold (M) cells are important in the mucosal immune system on intestine. Unlike other epithelial cells, M cells do not have a mucus layer on their apical side, thus facilitating antigen uptake on intestine [26]. M cells also facilitate the transport of *L. lactis* and its associated antigens to underlying immune cells, especially dendritic cells [70].

*L. lactis*-based vaccine and antigens captured by M cells and dendritic cells (APCs) on intestine are transported through the process of transcytosis to the basal side, such as Peyer's Patches (PPs). The APC cells phagocytize and internalize antigen and *L. lactis*-based vaccine along with its protein components. Antigens are presented by MHC-I and MHC-II molecules, presenting them to T and B cells, thereby triggering an adaptive immune response [18, 30, 32, 37]. Induced B cells differentiate into plasma cells, specifically prepared to produce antibodies. This immunological interaction involves CD4 T cells (Th cells), which send necessary signals through cytokines to promote class-switch recombination in B cells, leading to IgA production [71, 72]. These IgA-producing plasma cells then migrate to the lamina propria, where they continue to secrete dimeric IgA. Subsequently, IgA binds to the polymeric immunoglobulin receptor (pIgR) on epithelial cells, facilitating its translocation across the cell and its eventual release into the lumen as secretory IgA (sIgA). The region between the follicles around the PPs, called the Intrafollicular Region (IFR), is rich in T cells and dendritic cells and regulates the adaptive immune response. Through this mechanism, vaccine antigens carried by *L. lactis* will be presented and generate both innate and adaptive immune responses[73-76].

The IgA antibodies produced by *L. lactis*-based vaccinations significantly impact mucosal immunity. Mucosal IgA antibodies are crucial in the initial defense against infections that enter the body via mucosal surfaces. Fecal IgA antibodies are used as markers of secretory IgA in the gastrointestinal tract, providing valuable insights into the immune response associated with the gut. Moreover, Immunoglobulin A (IgA) antibodies found in nasal tissue and nasal fluid can also indicate immunity in the respiratory mucosa. Tissue IgA measurement can yield insights into the localization of specific IgA within tissues [77, 78]. IgA levels can be assessed by analyzing samples of feces, tissue (such as the gut, colon, and nose), and nasal fluid, as shown in Table 1. For example, oral vaccination with *L. lactis* expressing antigens from pathogens such as *H. pylori* or the *Influenza* has increased IgA antibodies, contributing to protective immunity [29-31]. This also proves that IgA can be formed to defend against pathogens like bacteria or viruses.

*L. lactis*-based vaccine can stimulate humoral immune responses of IgG antibodies. Studies in Table 1 have shown that vaccination based on *L. lactis* expressing antigens can increase significant IgG antibody responses [29, 37, 52]. In Table 1, oral vaccination based on *L. lactis* expressing antigens from pathogens such as *H. pylori* [15, 30, 31, 37], *Influenza* [19], [39], *Bordetella pertussis* [25], and *HIV-1* [29] has been shown to induce IgG antibodies contributing to humoral immunity.

In producing IgG or IgA antibodies, *L. lactis*-based vaccine are recognized by APCs in the mucosa. Dendritic cells present the antigen to CD4 T cells via MHC-II. CD4 T cells release cytokines such as IL-4 (Interleukin-4) and IL-6 (Interleukin-6), which are then responded to by B cells [25, 36]. This interaction allows B cells to mature into plasma cells. With the help of IL-4 and IL-21 from Th2 cells, plasma cells are prepared to produce IgG antibodies [79-82]. Additionally, class switching in plasma cells for IgA production occurs under the influence of TGF-β, IL-21, and IL-17 [83-86].

IgG antibodies can specifically bind to the pathogen antigen that triggers their production, thus forming an antigen-antibody complex. This facilitates pathogen recognition and elimination. IgG antibodies also neutralize pathogens, thereby enhancing the phagocytic response. Phagocytes, such as macrophages, have Fc receptors that bind to the Fc portion of IgG antibodies, triggering phagocytosis of pathogens opsonized by IgG antibodies[24, 37]. Additionally, IgG antibodies can activate the complement system, leading to the formation of a complex that damages pathogen membranes and triggers cell lysis [31, 32]. IgA antibodies can prevent pathogen adhesion to the mucosal surface through neutralization. The IgA antigen-antibody complex can be captured by immune cells such as macrophages and dendritic cells in the mucosa, which can then destroy and remove the complex from the body. In the digestive tract, the IgA antigen-antibody complex is secreted through feces [87, 88].

*L. lactis*-based vaccines can also induce cellular immune responses. Table 1 shows that not all studies report an increased cellular immune response; however, significant increases were observed in the studies by [26, 27, 29]. Vaccination using *L. lactis* -based vaccines increases dendritic cell activation by measuring MHC-II expression, CD4 T cells, CD8 T cells, and plasma cells by measuring CD138 expression. Dendritic cells play a critical role in initiating and modulating the cellular immune response. These cells function as antigen-presenting cells (APCs) by processing antigens and presenting them on major histocompatibility complex (MHC) molecules to T cells, activating the immune response. When vaccines utilizing *L. lactis* are administered, they deliver antigens directly to the host’s immune system. Upon administration, these bacteria or their components are internalized by dendritic cells. The antigens from the bacteria are then processed and presented via MHC-II molecules on the surface of the dendritic cells. Vaccines based on *L. lactis* enhance the expression of MHC-II molecules on dendritic cells. This augmented expression improves the capacity of dendritic cells to present antigens to CD4 T cells, thereby potentiating the immune response. As MHC-II molecules present antigens, CD4 T cells are more effectively activated. These activated CD4 T cells subsequently aid in activating B cells, resulting in antibody production and cytotoxic T cells, which can destroy infected cells [89, 90].

The antigens expressed by *L. lactis* extracellularly are broken down into smaller peptide fragments. Dendritic cells play an active role in this process. After capturing antigens, dendritic cells migrate to the lymph nodes, where they further process the antigens into smaller fragments and load them onto MHC-I molecules, essential for CD8 T cell activation. MHC-I molecules on the surface of dendritic cells interact with TCRs on naive CD8 T cells, leading to CD8 T cell activation. CD8 T cells then differentiate into cytotoxic T lymphocytes (CTLs). CTLs leave the lymph nodes and patrol the body, seeking cells that express the same antigen presented by *L. lactis*. Upon finding infected cells, CTLs release perforin and granzymes, which destroy the target cells [91, 92].

Evaluation of vaccine efficacy in animal models is critical. This is typically done through a challenge test involving exposing vaccinated experimental animals to pathogens. This test can determine the vaccinés ability to prevent infection or reduce the severity of the disease caused by the infection [15, 34]. Vaccine efficacy evaluation parameters include measuring the number of pathogens, clinical symptoms, immune responses, and survival rates in experimental animals, providing valuable information about the vaccinés effectiveness [15, 34]. A common challenge test assessment is histopathological evaluation, which determines the number of bacteria or viruses used to evaluate vaccine efficacy [93, 94]. Table 1 has several methods of evaluating vaccine efficacy other than immunologically. These evaluation methods include measurement of viral titer (viral load) [18, 19, 23], and virulence factor measurement [30, 31, 37]. Challenge tests can provide an in-depth understanding of the effects of vaccines on the immune system and disease development. Studies of challenge tests on mucosal vaccines administered orally or intranasally have shown that the immune response plays a vital role in protecting against infection by pathogenic microorganisms [93].

## Conclusion

*L. lactis* is suitable as a vector carrier for oral or nasal mucosal vaccines for bacterial and viral infections. *L. lactis*-based vaccine can induce cellular and humoral immune responses that protect against these infections. Research related to *L. lactis* as a mucosal vaccine carrier has great potential to continue to be carried out and developed.

## Figures and Tables

**Fig. 2 F2:**
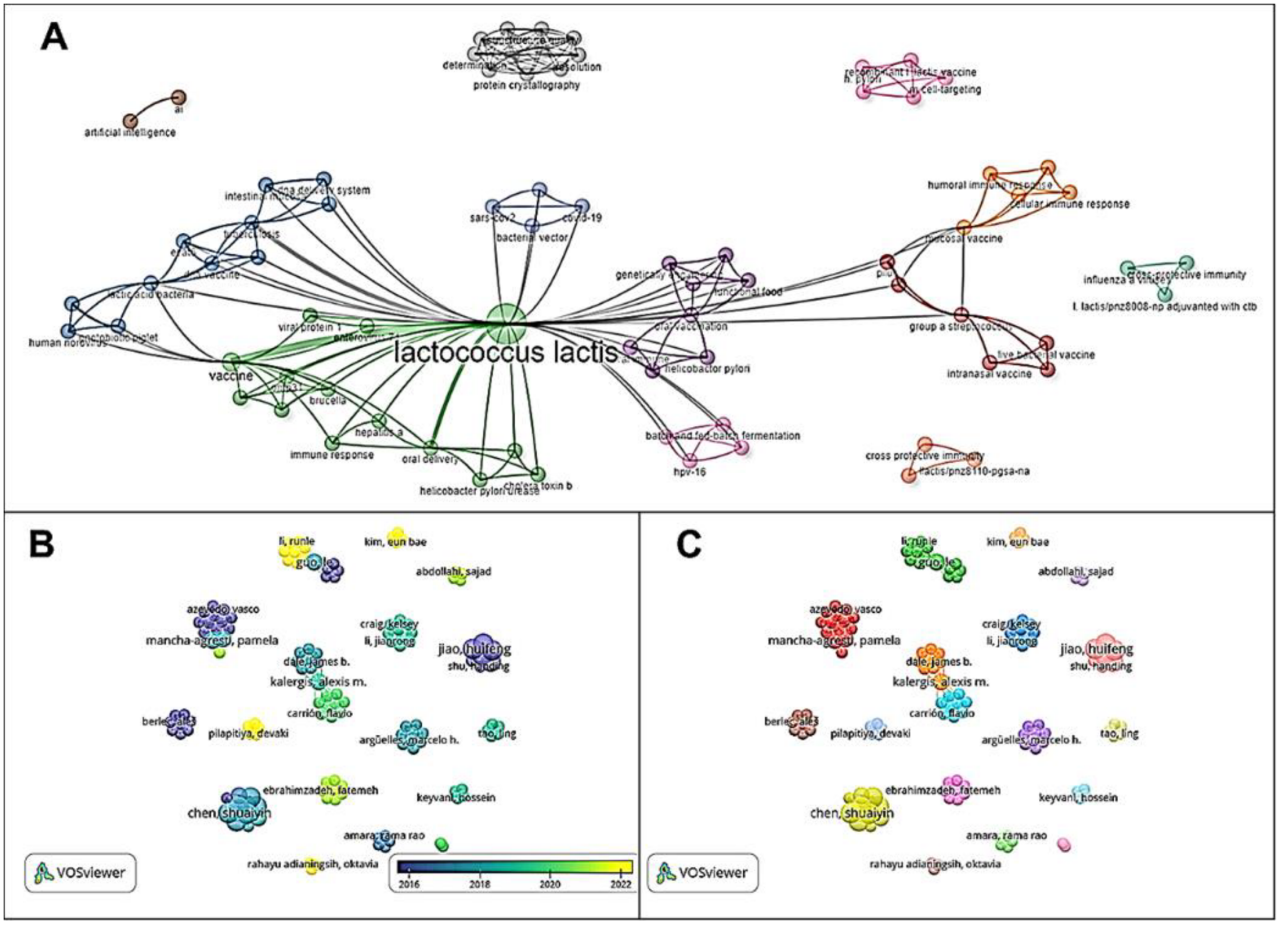
(A) Thematic Map, (B) Author Characteristics by Year and (C) Relationship Between Authors. (**A**) shows that *L. lactis* has been extensively studied as a mucosal vaccine carrier with various keywords connected to *L. lactis*. (**B**) Author Characteristics by Year shows the analysis of authors connected to *L. lactis*-based vaccine. This result shows that the research topic on *L. lactis* as a mucosal vaccination carrier continues to be carried out and develops yearly. (**C**) Relationship Between Authors shows the pattern of collaboration and research networks between authors on *L. lactis*-based vaccine. There are 130 authors and co-authors, but only 18 researchers are directly related to each other. Research on *L. lactis* as a mucosal vaccination carrier has been widely reported in various countries. These highlight the promising potential for developing *L. lactis* as a mucosal vaccine carrier.

**Table 1:** Characteristics of The Studies Included.

No	Author	Study models	Dosage (CFU)	Humoral immunity	Cellular Immunity	Cytokines	Challenged Test	*L. lactis* Strain	Plasmid
1	(Sun *et al*., 2019) [15]	BALB/c mice	1 × 10^11^ CFU	↑ IgG (Serum) & sIgA (Faecal)	-	-	-	NZ3900	pNZ8149
2	(Craig *et al*., 2019) [16]	Piglets	10^9^-10^12^ CFU	↑ IgG (Serum) & sIgA (Faecal)	-	-	-	NZ9000	pNZ8150
3	(Zhang *et al*., 2016) [17]	BALB/c mice	5 × 10^14^	↑ IgG (Serum) & sIgA not significantly increased (Faecal)	-	-	-	NZ3900	pNZ8119
4	(Lei, Peng, Jiao, *et al*., 2015) [18]	BALB/c mice	1 × 10^12^	↑ IgG (Serum) & sIgA (Intestine)	-	↑ IFN-γ & IL-4 (Splenocytes)	↓ *Influenza* titre (Lung)	NZ9000	pNZ8008
5	(Lei, Peng, Zhao, Jiao, *et al*., 2015) [19]	BALB/c mice	1 × 10^12^	↑ IgG (Serum), IgA (Intestine)	-	-	↓ *Influenza* titre (Lung)	NZ9000	pNZ8008
6	(Pereira *et al*., 2015) [20]	BALB/c mice	1 × 10^8^	↑ sIgA (Colon tissue, (Serum), & faecal)	-	↑ IFN-γ, TNF-α & IL- 12 (Spleen)	-	MG1363 FnBPA+	pValac
7	(J-Khemlani *et al*., 2023) [21]	FVB/n mice	1 × 10^8^	↑ IgG & IgA ((Serum), BAL, faecal & nasal)	-	-	-	MG1363	pLZ12-Km2 P23R
8	(Mohseni *et al*., 2019) [22]	C57BL/6 mice	1 × 10^9^	↑ IgG (Serum) & IgA (Vaginal fluid)	-	↑ IL-2 & IFN-γ (Splenocytes & intestinal)	-	NZ9000	pNZ8123
9	(Lei, Peng, Zhao, Ouyang, *et al*., 2015) [23]	BALB/c mice	1 × 10^12^	↑ IgG (Serum) & IgA (Intestine washes & upper respiratory washes)	-	-	↓ *Influenza* virus titre (Lung)	NZ9000	pNZ8110
10	(Castro *et al*., 2021) [24]	BALB/c mice	1 × 10^8^	↑ sIgA (Colon)	-	↑ IFN-γ, TNF-α, & IL-17 (Splenocytes)	-	MG1363 FnBPA+	pValac
11	(Torkashvand *et al*., 2018) [25]	BALB/c mice	1 × 10^8^	↑ IgG (Serum) & IgA (Lung)	-	↑ IFN-γ (Spleen)	-	NZ3900	pNZ8149
12	(Diaz-Dinamarca *et al*., 2020) [26]	C57BL/6 mice	1 × 10^10^	↑ IgG (Serum), IgA (Faecal & intestine)	↑ dendritic cell (CD45+, MHC-II+; CD103+, CD11c+, CD11b) and (CD45+, MHC-II+; CD11b+, CD103-)	-	↓ Group B *Streptococcus* colonization (Vaginal tract)	NZ9000	pNZ8124
13	(Yurina *et al*., 2023) [27]	BALB/c mice	5 × 10^9^ (Oral)/1 × 10^9^ (Nasal)	↑ IgG (Serum), IgA (Serum)	↑ plasma cell (CD138), CD4, and CD8	-	-	NZ3900	pNZ8149
14	(Xuan *et al*., 2022) [28]	BALB/c mice	3 × 10^10^	↑ IgG (Serum), IgA (Fecal)	-	-	-	IL1403	pILPtuf.Mb
15	(Chamcha *et al*., 2015) [29]	BALB/c mice	5 × 10^9^	↑ IgG (Serum & faeces) & IgA & (Serum, faeces, & vaginal wash)	↑ CD8 & dendritic cell (CD11b+ CD8α−)	-	-	MG1363	pJRS9550
16	(Li *et al*., 2014) [30]	BALB/c mice	2 × 10^9^	↑ IgG (Serum), IgG1 (Serum), IgG2 (Serum), IgA (Faecal)	-	-	↓ Urease *Helicobacter pylori* activity	NZ9000	pCYT, pMG & pHJ
17	(Peng *et al*., 2018) [31]	BALB/c mice	5 × 10^10^	↑ IgG (Serum) & SIgA (Faecal)	-	↑ IL-2. IFN- γ. IL-8, IL-10. IL-12, IL-17, IL-2, & IL-4	↓ Urease *Helicobacter pylori* activity	NZ3900	pNZ8110
18	(Ahmadi Rouzbahani *et al*., 2021) [32]	New Zealand rabbits	5 × 10^9^	↑ IgG (Serum) & IgA	-	-	↓ Enterotoxigenic *Escherichia coli* CFU	NZ3900	pNZ8149
19	(Wozniak *et al*., 2018) [33]	BALB/c mice	1 × 10^9^	↑ IgG (Serum & BAL)	-	-	↓ *Streptococcus pyogenes* colonization	NZ3900	pNZ8149
20	(Shirdast *et al*., 2021) [34]	BALB/c mice	1 × 10^10^	↑ IgM (Serum), IgG (Serum), IgA (Mucosa & serum)	-	-	-	NZ9000	pNZ7021
21	(Xu *et al*., 2019) [35]	BALB/c mice	1 × 10^9^	↑ IgG (Serum), IgA (Faecal)	-	-	-	MG1363	pMG36e
22	(Temprana *et al*., 2018) [36]	BALB/c mice	No Information	↑ IgG dan IgA	-	-	↓ Rotavirus shedding	NZ9000	No information
23	(Guo *et al*., 2022) [37]	BALB/c mice	1 × 10^10^ & 3 × 10^9^	↑ IgG (Serum) and sIgA (intestine, stomach, faeces)	-	↑ IFN-γ, IL- 4, IL, 17	↓ qPCR, CFU, Urease activity, & gastritis score *Helicobacter pylori*	NZ9000	plSAM
24	(Berlec *et al*., 2013) [38]	BALB/c mice	2 × 10^10^	↑ IgG (Serum) & IgA	-	-	-	NZ9000	pNZ8148
